# A Systematic Review on the Impact of Donor Characteristics and Donor Milk Handling on Infant Health and Growth

**DOI:** 10.1016/j.advnut.2026.100609

**Published:** 2026-02-19

**Authors:** Daniel Klotz, Agnieszka Bzikowska-Jura, Marzia Giribaldi, Magdalena Babiszewska-Aksamit, Tanya Cassidy, Laura Cavallarin, Serena Gandino, Karolina Karcz, Chiara Peila, Carolyn Smith, Bartłomiej Walczak, Aleksandra Wesołowska

**Affiliations:** 1 Bethel Center for Pediatrics, Department of Neonatology and Pediatric Intensive Care Medicine, University Hospital OWL, University of Bielefeld, Bielefeld, Germany; 2 Laboratory of Human Milk and Lactation Research, Department of Medical Biology, Medical University of Warsaw, Warsaw, Poland; 3 Institute of Sciences of Food Productions, National Research Council, Grugliasco, Italy; 4 Kathleen Lonsdale Institute for Human Health Research, Maynooth University, Maynooth, Ireland; 5 Nuffield Department of Women’s and Reproductive Health, University of Oxford, John Radcliffe Hospital, Oxford, United Kingdom; 6 Department of Neonatology, Wroclaw Medical University, Wroclaw, Poland; 7 Neonatal Intensive Care Unit, Department of Public Health and Pediatrics, University of Turin, Turin, Italy; 8 Bodleian Health Care Libraries, University of Oxford, Oxford, United Kingdom; 9 Institute of Applied Social Sciences, University of Warsaw, Warsaw, Poland; 10 Department of Clinical Dietetics, Medical University of Warsaw, Ciolka 27, 01-445 Warsaw, Poland

**Keywords:** donor human milk, infant, nutrition, outcome, growth, systematic review

## Abstract

Global recommendations advocate the conditional use of donor human milk (DHM), yet evidence on the influence of donor characteristics and DHM treatment on premature infants’ health and growth is lacking. This study aimed to compare clinical outcomes of infants fed standard DHM compared with modified DHM-based on predefined variations. We performed a systematic review searching 14 databases alongside clinical trial registries, up to 8 March, 2024. Any study designs, but case or animal studies, conference proceedings, reviews, and gray literature were included. Findings were synthesized narratively and in tables. Of 14,979 screened studies, 14 met inclusion criteria comparing clinical effects of DHM from mothers who gave birth prematurely compared with at term, DHM that was treated with high-temperature pasteurization methods compared with standard holder pasteurization, and concentrated or homogenized DHM compared with standard DHM. Our analysis revealed a significant paucity of clinical data for several interventions and a complete lack of evidence for most of our research questions, indicating insufficient evidence to support the clinical use and efficacy of the aforementioned interventions. We found substantial evidence gaps in understanding how DHM treatments influence health and growth in premature infants. Research prioritizing clinical outcomes is essential to guide milk-banking policies.

The protocol was registered on PROSPERO as CRD42024522015.

**Statement of significance**

This systematic review reveals a critical evidence gap in donor human milk research, finding that despite extensive preclinical data on milk composition variations, only 14 studies with 540 infants could be identified to assess how different donor characteristics and processing methods affect infant health outcomes. This represents the first comprehensive systematic review specifically examining clinical outcomes related to donor selection criteria and milk processing variations, uniquely mapping the complete absence of clinical evidence for most donor milk-banking interventions despite widespread preclinical research.

## Introduction

Donor human milk (DHM) is a natural substance of human origin obtained from breast milk that exhibits a range of organic qualities due to both inherent biological variation and treatment-related factors [1].

Biological variations of DHM arise from certain donor characteristics. Fixed and variable donor characteristics include the gestational age of the donor’s own offspring, the stage of lactation, and various lifestyle factors, including dietary habits and smoking behaviors [[2], [3], [4]]. For example, lower gestational age and earlier lactation stages are associated with an increased human milk (HM) protein content compared with later gestational age and lactation stage [5,6].

Treatment-related factors include testing procedures, methods, and equipment used for the collection, processing, and storage of DHM, which differ considerably between facilities [7]. For example, various treatment methods, including thermal treatments such as heating [holder pasteurization (HoP)] and freezing DHM, can significantly influence the composition of DHM [[8], [9], [10]]. Similarly, variation in donor hygiene practices during expression and processing can influence the bacterial contamination levels of DHM, yet no established consensus currently exists regarding optimal screening schedules or microbiological criteria for defining milk acceptability before and after pasteurization [8].

Both biological and treatment-associated variations in DHM may affect recipient health and growth outcomes. Previous reviews have addressed the impact of these interventions on DHM quality and revealed an abundance of preclinical data on milk composition and safety aspects [[9], [10], [11], [12], [13]]. No systematic review is available about the clinical influence of the above-mentioned variations of selecting DHM donors, collecting DHM, or processing DHM on the actual health and growth outcomes of infants compared with DHM donated or processed using established standard protocols.

The present study aimed to systematically evaluate the evidence regarding the influence of these factors or interventions during donor selection, collection, or processing on any clinical outcomes of infants fed with DHM, including growth, development, cognitive functions, health outcomes, rates and types of infections, and micronutrient deficiencies.

## Methods

### Research question

We used a modified Population, Intervention, Comparison, Outcome (PICO) format to structure the review. The PICO format was determined by the WHO Guideline Development Group on DHM Banking, aiming to establish global standards on minimum requirements of safety and quality, with guidance addressing all aspects of HM banking [14]. The PICO criteria are given in Table 1.

**TABLE 1:** The modified PICO criteria

Parameter	Inclusion criteria
Population	Are infants receiving DHM (exclusively or in conjunction with MOM or with breast milk substitutes and irrespective of the actual amount of DHM received)
Intervention 1	When donors are characterized by different lactation stages
Intervention 2	When donors are characterized by different birth outcomes (i.e., delivery at term or prematurely)
Intervention 3	When donors are characterized by different dietary regimens
Intervention 4	When donors are characterized by different lifestyle factors (e.g., smoking, increased infectious or toxicological risks)
Intervention 5	When donors are characterized by different health status (e.g., acute or chronic illness, BMI outside healthy limit)
Intervention 6	When donated breast milk is expressed with different methods
Intervention 7	When different hygiene practices or settings are used during donated milk expression
Intervention 8	When donated milk is treated with different methods (e.g., pasteurization, homogenization, increase of energy density)
Intervention 9	When different testing procedures are used on donations
Outcome	Differently affected in their growth, development and/or health status
Study design	Any (excluding case reports)

Abbreviations: DHM, donor human milk; MOM, mother’s own milk, PICO, Population, Intervention, Comparison, Outcome.

The population of interest comprises infants receiving DHM, which differed from conventionally processed DHM in 9 predefined interventions based on donor selection, screening procedures, or processing methods (Table 1). However, the comparison element was not defined a priori, to allow inclusion of studies assessing varying levels of implementation, comparisons across multiple interventions, or the absence of an intervention altogether. Clinical outcomes included any outcomes that were determined in the infant such as growth parameter assessment, morbidity [including bronchopulmonary dysplasia (BPD), necrotizing enterocolitis (NEC), retinopathy of prematurity (ROP), patent ductus arteriosus (PDA), intraventricular hemorrhage (IVH)], mortality (any timepoint and cause), feeding tolerance during hospital stay (defined by duration of parenteral nutrition, time to full enteral feeding or predefined feeding intolerance score). Also included as clinical outcomes were adverse events (any unwanted medical occurrence in DHM receiving patients, regardless of whether it is related to the intervention or not), infections (any etiology), nutritional deficiencies (vitamins and/or micronutrients), biochemical studies (biochemical serum concentrations), and neurodevelopmental outcomes (any neurodevelopmental assessment).

We incorporated any reported variations of health and growth outcome parameters to ensure inclusivity across all relevant outcome variables. In case there was no direct and equivalent comparator, e.g., term compared with preterm DHM, we decided to include studies only if a comparison to a (retrospective) control group could be made or if specific or recommended standard reference ranges were available (e.g., WHO growth charts). According to our research question, we did not include any comparisons of DHM with mother’s own milk (MOM) or breast milk substitutes (BMS).

### Search strategy and selection criteria

We conducted a systematic review including heterogeneous trials with no summary estimate in line with PRISMA guidelines [15] (Supplementary Table 1). The study was prospectively registered on PROSPERO [16], and the protocol was published in an open-access journal [17].

We searched Global Index Medicus, CINAHL on Ebscohost, Medline, Embase, Emcare, PubMed, Global Health on OVID, Google Scholar, Scopus, and the Web of Science Core Collection. For recently completed or ongoing trials, we searched CENTRAL, clinicaltrials.gov, WHO ICTRP, and the EU/EEA Clinical Trials Register. The Boolean strategy consisted of search terms on the key concepts of “donated human milk” and “effect on infants” and was adapted for the individual bibliographic databases (Supplementary Table 2). In a hybrid, snowballing search strategy, we hand searched PubMed and Google Scholar for reference lists, “cited by” and “similar articles,” restricted to the 10 most cited articles found in the main search and to the first 200 records found in this way. The reference list of relevant reviews was also screened manually to identify potentially pertinent additional studies.

Institutional review board approval was not required because we did not collect original data.

The search was carried out on 8 March, 2024. The search results from different databases were imported into a systematic review software (Covidence, Veritas Health Innovation). Duplicates were deleted by the review software and the reviewers during the screening process. Two reviewers independently screened titles and abstracts and performed the full-text review. Discrepancies were resolved with a third researcher. Data were independently extracted in Covidence in a predefined data extraction template for study characteristics, participants, interventions, and any outcomes, disparities were discussed within the entire study team until consensus was achieved.

We included any eligible published or unpublished studies provided that an abstract was available in English, irrespective of the study design, except case studies. We did not apply any setting, time frame, or language restriction. Translations of non-English manuscripts were performed using a large language model (DeepL Translate, DeepL SE).

We excluded gray literature, conference proceedings, animal studies, and publications with no original data—including editorials, comments, letters, book chapters, and reviews. According to our research question, we did not include any studies on clinical outcomes comparing MOM with DHM. We contacted authors of abstracts and principal investigators of published study protocols to ascertain whether further results might be available and eligible, integrating, when possible, available information on extracted data. In addition, we excluded interventions that used proprietary technologies when manufacturing details or application methods were not publicly available.

### Quality assessment and grading of recommendations assessment, development, and evaluation

Quality assessment of all included studies was carried out by 2 authors separately using the revised Cochrane risk of bias (RoB) tool for randomized trials (RoB 2.0 tool) [18] (see Supplementary Table 3) and Critical Appraisal Skills Program [19] for observational studies (see Supplementary Table 4). Any discrepancies were resolved through discussion, and the full process is presented in Supplementary Figure 1.

We scored the “Transparency of the Methodology and Results” for both randomized controlled trials (RCTs) and Observation Studies, categorizing them either as yes (coded 2), no (coded 0), or unsure (coded 1). We then created an overall summary score for each study, which we grouped into potential quartiles by dividing the possible potential scores, determining the following categorical classifications of the highest or fourth quartile being labeled Excellent, Good —the third quartile, Fair—the second quartile range, and Poor refers to scores within the first quartile range regarding transparency. Regarding the Bias classification, we determined Low (green) bias to be the categories of either Excellent or Good levels of transparency, and High (red) bias to be the categories of either Fair or Poor exclusively. If there was a combination of Excellent/Good and Fair/Poor, the classification for Transparency and Bias, we determined Some (yellow) as the final category. The complete scores can be viewed in the Supplementary Tables 3 and 4, with some categories not being applicable to some articles. Quartiles were determined from each article using these scores. We then created a RoB score for each study using the standard terms and colors Low (green), Some (yellow), and High (red) (Supplementary Figure 2).

We developed a Cochrane Effective Practice and Organization of Care (EPOC) review worksheets [20], determining the confidence of the results in 5 dimensions: publication bias, inconsistency, indirectness, imprecision, and RoB (based on the quality assessment of the methodology and results from the quality assessment for each individual study discussed above). Inconsistency and indirectness were judged by 2 clinicians, and the other characteristics by 2 methodologists. Two reviewers assessed each dimension using a 3-point rating system: “not at all serious,” “not very serious,” and “serious” (Supplementary Table 5). Following EPOC [20] rules: initial confidence was 4 points for RCT, 2 points for nonrandomized studies, and a determination (not at all, not very serious, extremely serious) was given for each of the 5 dimensions (RoB, inconsistency, indirectness, imprecision, publication bias). No change was given for the grade of “not at all,” –1 was given for a grade of “not very serious,” for a “serious” grade –2 points were given for each of the 5 dimensions. These dimensions were then used to create an overall grading of recommendations assessment, development, and evaluation using standard labels “high certainty” (meaning a grading of “not very serious” across all dimensions, resulting in reporting very confident that the true effect is close to the estimate), “moderate certainty” (meaning a grading of “not very” or “serious” on 1 dimension, resulting in moderate confidence), “low certainty” (meaning a grading of “not very serious” or “serious” on 2 dimensions resulting limited confidence), and “very low certainty” (meaning a grading of “not very serious” or “serious” on 3 or more dimensions, resulting in very little confidence in the effect estimate the true effect).

### Data analysis and synthesis

Due to the heterogeneity of the included studies, we did not perform a quantitative meta-analysis but provided results in summary tables and qualitative narrative synthesis and grouped outcome criteria to address evidence gaps.

## Results

### Study selection

**FIGURE 1:**
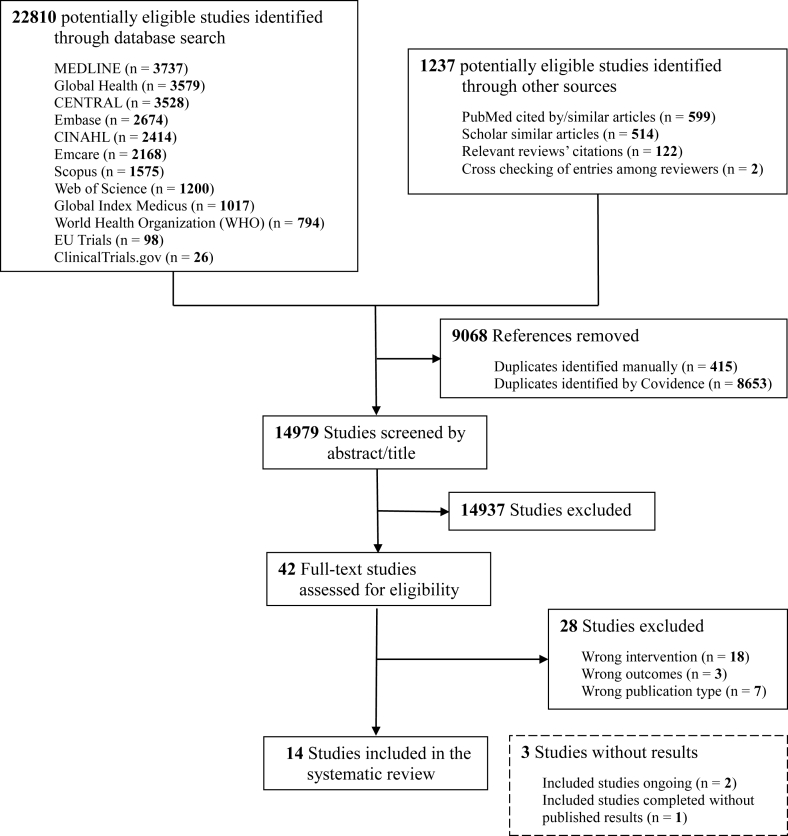
PRISMA flowchart for the systematic review.

We identified 22,180 records from database searches, resulting in 14,979 unique records for screening. Of these, 42 records were found to be relevant for full-text screening. Ultimately, 14 records met the eligibility criteria and were included in the final analysis (Figure 1). The reasons for excluding records in the full-text analysis are given in the Supplementary Table 6.

### Overall characteristics of studies included in the review

In total, we identified and included 14 studies for intervention 2 (gestational age at birth, *n* = 3), intervention 5 (donor characterized by different health status, *n* = 1), and intervention 8 (donor milk treated with different methods, *n* = 10). We were not able to identify any relevant studies for the remainder of the reviewed interventions. We retrieved 11 RCTs and 3 observational studies.

**FIGURE 2:**
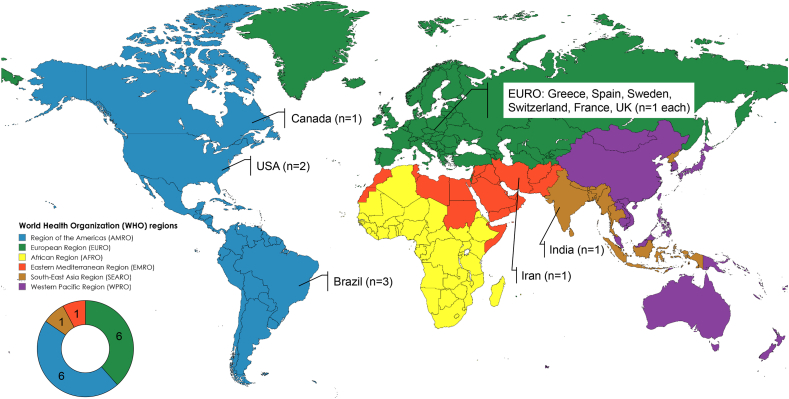
Map indicating the location of the included studies (*n* = 14).

The basic characteristics of the included studies are given in Table 2 [[21], [22], [23], [24], [25], [26], [27], [28], [29], [30], [31], [32], [33], [34]]. For each included study, we only stated the number of enrolled infants that exclusively received DHM because some strata or subgroups within the included studies contained MOM or BMS as intervention. A total number of 540 infants were enrolled in the included studies, which covered a period from 1952 to 2024, originating from 11 different countries and 4 different WHO regions (Figure 2).

**TABLE 2:** Basic characteristics of the included studies

Authors^1^	Study design	Setting (country, y)	Sample size (*n*)^2^	Population (infants gestational age)^3^	Interventions^2^	Control/comparisons		Outcomes^4^
**Intervention 2: donors are characterized by different birth outcomes**								
Gialeli et al. 2023 [21]	Single-center parallel RCT	Greece 2017–2021	128	Extremely to very preterm	Preterm DHM	Term DHM		Protein and caloric intake, Somatic growth, culture-positive sepsis, NEC, ROP, BPD, IVH, PDA
Gross 1983 [22]	Single-center parallel RCT	USA 1980–1982	40	Very preterm	Preterm DHM	Term DHM		Somatic growth, serum electrolytes, total protein and albumin concentration, calcium-phosphate and acid-base homeostasis
Soni et al. 2022 [23]	Single-center parallel RCT	India 2020–2021	102	Very to moderate preterm	Preterm DHM	Term DHM		Days to regain birth weight, somatic growth, days to full enteral diet, feeding intolerance, NEC
de Oliveira et al. 2017 [24]	Single-center crossover RCT	France 2014–2015	8	Very preterm	Homogenized DHM	DHM		postprandial gastric volume lipolysis, proteolysis and structural disintegration of DHM
dos Santos et al. 2007 [25]	Single-center parallel RCT	Brazil 2000–2002	20	Very to moderate preterm	Concentrated DHM	DHM		Plasma amino acid concentrations, somatic growth
García-Lara et al. 2024 [26]	Multicenter parallel RCT	Spain 2020–2023	160	Extremely preterm	HTST DHM	HoP DHM		Catheter-associated sepsis, NEC, BPD, ROP, mortality, feeding intolerance, somatic growth, length of hospital stay, type of feeding at discharge
Nars 1984 [27]	Single-center prospective cohort study	Switzerland	18	Very preterm	Concentrated DHM	DHM (historic control group, *n* = 57)		Somatic growth, serum electrolytes and urea concentration, aminoaciduria, NEC, PDA
Rayol et al, 1993 [28]	Single-center parallel RCT	Brazil	30	Very to moderate preterm	Homogenized DHM	DHM		Somatic growth, fat balance
Söderhjelm 1952 [29]	Multicenter cohort study	Sweden	21	Extremely low to low birth weight	Various DHM heat treatment protocols	—		Fat balance
Thomaz et al. 2014 [30]	Single-center parallel RCT	Brazil 2008–2010	14	Extremely to very preterm	DHM fortified with evaporated HM	DHM fortified with lyophilized HM		Phenylalanine plasma concentrations
Williamson et al. 1978 [31]	Multicenter crossover RCT	United Kingdom	7	Extremely to moderate preterm	Nonpasteurized DHM flash-heated DHM	DHM		Fat, nitrogen, sodium, calcium and phosphate balance
**Clinical trials without available results**								
**Intervention 5: donors are characterized by different health status (e.g., acute or chronic illness, BMI outside healthy limit)**								
Azad 2019 [32]	Multicenter Parallel RCT^5^	Canada 2023–ongoing		60	Extremely to moderate preterm	HMO secretor status-matched DHM	DHM	Fecal microbiome composition, somatic growth, days to full enteral diet, length of hospital stay
**Intervention 8: donor milk is treated with different methods (e.g., pasteurization, homogenization, increase of energy density)**								
Hemati 2024 [33]	Multicenter Parallel RCT^5^	Iran 2024–ongoing		124	Extremely to moderate preterm	DHM pasteurized in plastic container	DHM pasteurized in glass container	Estrogen serum concentrations, thyroid serum concentrations, growth hormones and IgA concentrations, somatic growth
Perry 2020 [34]	Single-center prospective cohort study	United States 2019–2020		31	Late preterm to term infants	UHT pasteurized DHM	WHO growth chart	Somatic growth, rates of exclusive HM diet

Infants’ gestation is given as mean ± SD or median (IQR) where applicable.

Abbreviations: BPD, bronchopulmonary dysplasia; DHM, (pasteurized) donor human milk; HM, human milk; HMO, human milk oligosaccharides; HoP, holder pasteurized; HTST, high-temperature short-time (pasteurized); IVH, intraventricular hemorrhage; NICU, neonatal intensive care unit; NEC, necrotizing enterocolitis; PDA, patent ductus arteriosus; RCT, randomized clinical trial; ROP, retinopathy of prematurity; UHT, ultrahigh-temperature.

1 For ongoing or terminated clinical trials without published data, or clinical trials with unknown status date of first study registration is given.

2 In case of multiple parallel arms and dropouts, only the final sample size and intervention for study arms involving DHM are reported in this table.

3 According to the WHO-definition.

4 Primary study outcome listed first.

5 No data published, information according to published study protocol.

We identified 3 clinical trials without published results. Two of those are still actively enrolling patients [32,33], whereas the third trial is registered as completed [34]. Primary outcomes of these trials are fecal microbiome composition based on donor HM oligosaccharides (HMO) secretor status [32] (intervention 5, donor health status), estrogen and thyroid serum concentrations in infants receiving DHM that were stored in plastic compared with glass containers [33], and somatic growth rates in infants that received ultrahigh-temperature pasteurized DHM [34] (both intervention 8, DHM treatment). Data for these trials are not available for integration in our review, either due to personal communication indicating data unavailability (*n* = 1) or to a lack of response from the principal investigators (*n* = 2).

**FIGURE 3:**
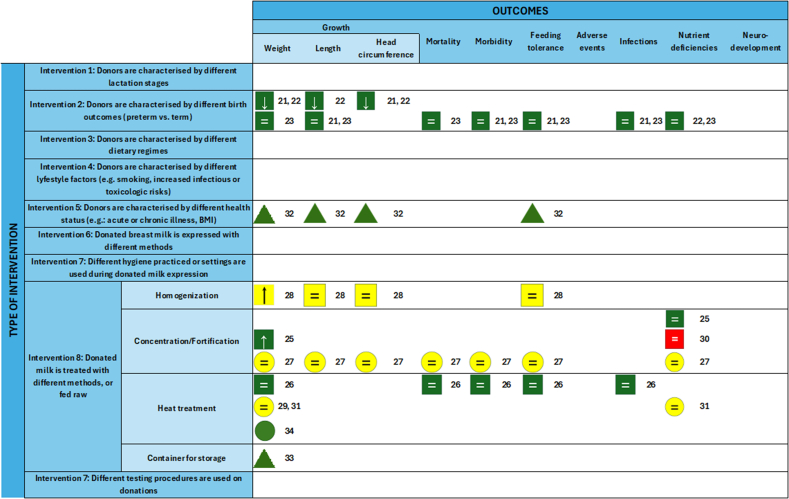
Evidence Gap Map of reviewed clinical outcomes. Each icon represents an outcome reported in an individual study. The numbers correspond to the reference list of the selected full-text articles. Green square: high-quality RCT; yellow square: moderate-quality RCT; red square: low-quality RCT; green circle: high-quality observational; yellow circle: moderate-quality observational; triangle: ongoing study; **↑**: increased by intervention; =: unaffected by intervention; ↓ decreased by intervention. RCT, randomized controlled trial.

Eleven of 14 studies were published and included outcome categories for intervention 2 (gestational age at birth) and intervention 8 (DHM treatment). Intervention 2 consistently compared DHM from mothers giving birth prematurely to those giving birth at term. Intervention 8 encompassed a comprehensive range of milk treatment methodologies for evaluation before consumption. These treatment methodologies are discussed in detail below. However, for the majority of the specified outcome criteria, we were unable to identify any evidence from the published literature, as illustrated by the evidence gap map (Figure 3).

### Results on the effects of specific DHM interventions

#### Intervention 2—donors who delivered at term or prematurely

Regarding intervention 2, the primary outcome was to compare the infants’ health and growth outcomes when fed with DHM from mothers who delivered their infants either preterm or at term (Table 3).

**Table 3:** Detailed data of included and published studies

Study	Donor human milk characteristics/treatment	Infants characteristics at inclusion	Intervention Diet	Control diet	Outcomes	Result (intervention vs controls)	p-value
**Intervention 2 - Preterm Domor Human milk (Donor has given birth to a preterm infant irrespective of the stage of lactation)**^a^							
Gross, 1983^22^	Pooled DHM from mothers who gave birth at < 35 wks of GA. DHM was pooled from several donors according to week of lactation, starting after delivery Pooled term DHM from 3 to 12 months of lactation	30.6 ± 0.3 vs 31.0 ± 0.4 GA (wks), 1322 ± 54 vs 1321 ± 59 BW (g)	Preterm DHM 180 ml/kg/day until 1800 g	Mature DHM 180 ml/kg/day until 1800 g	Time to regain BW (d)	11.4 ± 0.8 vs 18.8 ± 1.7	<0.001
					Weight gain (g/d)	23.7 ± 1.1 vs 15.8 ± 0.8	<0.001
					Crown-to-heel length (cm/wk)	0.75 ± 0.03 vs 0.54 ± 0.04	<0.001
					Head circumference (cm/wk)	0.84 ± 0.03 vs 0.70 ± 0.02	<0.001
					Incidence of hyponatremia (%)	15 vs 50	<0.05
					Serum phosphorus	decreased in pDHM group	<0.005
Gialeli 2023^21^	Preterm DHM from mothers who gave birth at < 35 wks of GA for the first 4 weeks of lactation Term DHM up to six months lactation after delivery	29.7 ± 2.5 GA (wks), 1169.8 ± 234.1 BW (g)	Preterm DHM in the first 3 wks of life	Mature DHM in the first 3 wks of life	Protein intake in the 3rd wk donor milk period (g/kg/d)	3.37 ± 0.96 vs 3.19 ± 0.97	0.023
					Weight at discharge (g)	2560.7 ± 423.4 vs 2411.8 ± 370.5	0.047
Soni 2023^23^	MOM supplementation with “preterm DHM” MOM Supplementation with “term DHM”	31.3 ± 2.8 vs 31.8 ± 2.4 GA (wks) 1262 ± 184 vs 1253 ± 181 BW (g)	MOM supplemented with preterm DHM 180-200 ml/kg/d for a median of 6.5 d	MOM supplemented with term DHM 180-200 ml/kg/d for a median of 7 d	Time to regain BW (days)	17.4 ± 7.7 vs 13.6 ± 7.2	0.02
					Weight gain in 4^th^ wk (g/kg/d)	10.6 ± 4.0 vs 14.3 ± 3.9	<0.01
					Hypoglycemia (%)	2 vs 23	<0.01
**Intervention 8** – **high energy density milk studies**							
dos Santos 2007^24^	DHM evaporated by controlled evaporation to 70% of its initial volume using a vacuum rotating evaporator at 56°C under a mean vacuum pressure of 62.5 cm H_2_O	31 (28-34) to 32 (29-32) GA (wks), 11±1 d postnatal age	concentrated DHM 170 –180 mL/kg/d concentrated DHM for 10–12 days	non-concentrated DHM, otherwise idem	plasma amino acid concentrations	no difference	>0.05
					absolute weight gain during intervention (g)	188 vs 90	<0.01
Nars, 1984^26^	DHM supplemented with 40% lyophilisate solution of DHM human milk	29.8±2.8 to 29.9±5.0 GA (wks), ≤ 4 h postnatal age	supplemented DHM 130 mL/kg/d for 21 days	non-supplemented DHM (historic control group)	serum electrolytes (Na, Ca, Mg)	no difference	NR
					aminoaciduria, creatinuria	no difference	NR
					weight gain in 3^rd^ wk of life (g)	150 ± 46 vs 154 ± 45	NR
					NEC (n)	3.3 vs 3.5	NR
					PDA (n)	18 vs 27	NR
Thomaz 2014^30^	DHM supplemented with 20% evaporated DHM after lactose extraction and added 80 mL of pasteurized HM DHM defatted milk evaporated at 20% after lactose extraction, lyophilized and reconstituted in 100 ml DHM	26.6±2.9 to 30.0±1.3 GA (wks)	DHM supplemented with evaporated DHM for 15 ± 2 days	DHM fortified with lyophilized DHM for 15 ± 2 days	plasma phenylalanine levels (μmol/L)	13.44 ± 0.61 vs 15.42 ± 0.83	>0.05
**Intervention 8 – Heat treatment studies**							
García-Lara 2024^25^	HTST continuous flow pasteurized DHM	26 (25-27) to 27 (25-28) GA (wks)	HTST treated DHM from birth until 34 wks GA		catheter associated sepsis (%)	41.8 vs 45.7	0.62
					episodes of confirmed catheter-associated sepsis/1000 catheter days (n)	17.5 vs 18.7	0.78
					mortality (%)	5.1 vs 4.9	0.97
					surgical NEC (%)	6.3 vs 2.5	0.23
					NEC Bell grade ≥II or confirmed sepsis (%)	45.6 vs 48.2	0.74
					spontaneous intestinal perforation (%)	0 vs 2.5	0.16
					oxygen requirement at 36 wks GA (%)	26 vs 28.2	0.82
					ROP requiring treatment (%)	14.5 vs 18	0.54
					median hospital stay (d)	85 vs 79 d	0.95
					median weight gain birth to 36 wks GA (g/kg/day)	12.8 vs 12.7	0.33
Soderhjelm 1952^29^	DHM autoclaved at 114°C then frozen, 72°C/5 min, 97°C/3 min, 62°C/20 min, 97°C/20 min	960 to 2200 g at intervention	heat treated DHM at 140-190 mL/kg/d for 4-7 d	NA	Fat adsorption rate (%)	92 vs 100	NA
Williamson 1978^31^	DHM pasteurized at 63°C/30 min, milk heated at boiling point then	30.3±1.9 GA (wks)	pasteurized DHM at ∼250 mL/kd/d for 1 wk	boiled DHM at ∼250 mL/kd/d for 1 wk	Fat adsorption rate (%)	72.9 vs 62.4	0.05
					nitrogen absorption (%)	80.9 vs 81.5	NS
					electrolyte absorption (%) (Calcium, sodium, phosphorus)	no difference	NS
					mean weight gain (g/100 mL milk)	6.34 vs 6.63	NS
**Intervention 8 – Homogenisation studies**							
de Oliveira 2017^27^	Ultrasonic homogenization for 15 minutes	29.5±1.5 GA (wks)	homogenized DHM test meals over 6 d period	non-homogenized DHM	postprandial gastric volume (mL/kg)	11.0±3.6 vs 6.8±3	<0.01
					lipolysis level (%)	6.8±2.0 vs 3.2±1.2	<0.01
					protein digestion (%)	no difference	>0.05
Rayol 1993^28^	Ultrasonic homogenization for 15 minutes	31.9±2.5 to 32.6±1.6 GA (wks)	homogenized DHM for 20 d (fat balance over 3 days)	non-homogenized DHM	fecal fat loss (g)	2.7±1.7 vs 2.7±1.0	NS
					weight gain (g)	424.6±59.8 vs 349.3±74.9	<0.01

Values are reported as mean (SD), median (IQR) or median. Values were reported as “not significant” or as “no difference” as reported in the original study.

All DHM in included studies was Holder pasteurised if not indicated otherwise.

BW, birth weight; BL, length at birth; DHM, donor human milk; HM, human milk; MOM, mother’s own milk; NEC, necrotising enterocolitis; OFC, occipito-frontal circumference; PDA, patent ductus arteriosus; PDM; preterm donor milk, NA, not applicable; NR, not reported; NS, not significant; SGA, small for gestational age; TDM, term donor milk;

a Due to multiple outcomes only differing results are presented for intervention 2.

Gross [22] performed a single-center RCT enrolling 40 preterm infants with a birth weight of ≤1600 g. These infants were randomly assigned to receive either DHM from mothers who gave birth at <35 wk of gestation or to receive term DHM from up to 12 mo of lactation up to a body weight of 1800 g. Regaining birth weight was improved in the term milk group compared with the preterm DHM group, whereas daily weight gain, length, and head circumference were improved in the preterm DHM group.

Gialeli et al. [24] observed an increased protein intake and increased weight at discharge in 120 extremely preterm infants if they supplemented MOM with preterm DHM compared with term DHM for 4 wk postnatally (Table 3).

In their nonblinded RCT enrolling 102 preterm infants with a birth weight of ≤1500 g, Soni et al. [23] found a prolonged time to regain birth weight when MOM was supplemented with preterm compared with term DHM. However, weight gain was not different in the combined first 4 wk of life [median (IQR) 8.2 (4.6) compared with 9.3 (5.1), *P* = 0.33]. In this study, MOM was supplemented with preterm or term DHM for a median of 6.5 or 7 d, respectively (Table 3), but the overall proportion of each term or preterm DHM was <20%, and the proportion of MOM usage was significantly lower in the preterm milk group (82.0% compared with 91.1%, *P* < 0.03).

#### Intervention 8—donor milk treatment

The treatment modalities under investigation included DHM with increased energy density, homogenization of DHM, and the application of different heat treatment technologies and protocols. Detailed results are given in Table 3.

#### Increased energy density

Three studies [25,27,30] compared the use of DHM with an increased energy density in very to moderate preterm infants. In their parallel design RCT, dos Santos et al. [25] found no difference in overall plasma amino acid concentrations between very to moderate premature infants fed with concentrated DHM compared with standard DHM (Table 3). However, they found an increased weight gain in infants fed with concentrated DHM compared with those fed with standard DHM (90 g compared with 188 g, *P* < 0.01). The specific macronutrient and energy content of the respective milk was not analyzed [25].

In the single-center prospective cohort study, 18 very premature infants were fed with DHM fortified with a 40% DHM lyophilizate solution [27]. No difference was observed in any of the compared growth parameters and NEC or PDA rate (NEC 3.3% compared with 3.5%, point estimate not reported) when compared with a historical control group (Table 3).

Both strategies—condensation and the addition of lyophilized DHM—were compared by Thomaz et al. [30] in their RCT. They found no differences in phenylalanine plasma concentrations among 14 premature infants. This is relevant since elevated phenylalanine concentrations, typically found in bovine milk casein-based ingredients, can impair brain development through tyrosinase inhibition.

#### Heat treatment interventions

In their parallel design RCT enrolling 160 preterm infants, García-Lara et al. [26] found no difference in the rate of catheter-related sepsis [41.8% compared with 45.7%, *P* = 0.62, relative risk 0.92 (confidence interval: 0.68, 1.26)] between extremely premature infants fed DHM processed with a high-temperature short-time treatment and those fed holder pasteurized DHM. In addition, no differences were observed in the type of feeding at discharge, the number of episodes of feeding tolerance per infant, or any other secondary outcome criteria (Table 3). However, due to recruitment difficulties, this study did not reach its targeted sample size, and the overall volume of milk applied to the participants was 82.9% MOM and only 17.1% DHM.

In a small cohort study from 1952, the degree of fat absorption of 21 preterm infants was reported at 92% after being fed with DHM that was treated with different heat treatment protocols from 62°C at 20 min to 100°C for 3 min [29]. In this study, autoclaving DHM before freezing, followed by heat treatment, did not influence fat balance in a subgroup of 9 infants (Table 3).

However, fat absorption was reduced in a crossover trial including 7 preterm infants when HoP milk was compared with flash-heated feedings. The authors found no difference in nitrogen or electrolyte retention rates [31].

#### Homogenization

The clinical outcome of DHM homogenization was investigated by Rayol et al. [28] in a single-center parallel RCT enrolling 30 premature infants. They found no difference in fat balance but a greater weight gain after a 20-d intervention when infants were fed homogenized DHM (424.6 ± 59.8 g) instead of nonhomogenized DHM (349.3 ± 74.9 g; *P* < 0.01). The application of homogenized DHM test meals revealed an increase in postprandial gastric volume as well as in the gastric emptying rate in another crossover trial in 8 premature infants [24] (Table 3).

## Discussion

The advantages of DHM for premature infants compared with BMS are well documented and resulted in near-universal recommendations of varying strengths for the use of DHM if MOM is not available [35,36]. The present systematic approach was not designed to review the extant literature that compares DHM with BMS or with MOM, but to review growth and health outcomes of preterm infants that were exposed to DHM obtained from different donors or treated in different manners compared with conventional donor selection and processing. In this regard, and to the best of our knowledge, this is the first effort to comprehensively search the extant literature on this subject, presenting a thorough overview of studies assessing the impact of donor milk characteristics on infants’ growth and health outcomes.

### Evidence gaps

Our survey and evidence gap map reveal a critical shortage of studies examining clinical health and growth outcomes in premature infants related to HM composition variations and donor characteristics. This evidence gap is particularly striking given the wealth of available data on HM constituents under different conditions—including various pasteurization techniques, gestational ages, and lactation durations.

We identified notable gaps in understanding the impact of donor characteristics and donor milk handling on infant health and growth outcomes. It is evident from preclinical data that various predefined criteria could affect the composition and availability of DHM, potentially influencing neonatal and infant health. However, clinical studies are largely absent for most of these interventions. Although there are studies comparing MOM and DHM, these do not adequately address the specific effects of the interventions nor answer the original research questions.

The limited studies we did identify present additional challenges for drawing meaningful conclusions. These few investigations span over 7 decades and, although generally of satisfactory quality, show significant heterogeneity in their target populations, outcome measures, and intervention protocols. Furthermore, most studies lacked adequate statistical power and were designed primarily to generate hypotheses rather than provide definitive evidence. The available evidence, predominantly derived from small studies, is insufficient to inform clinical practice with high confidence.

### Intervention 2

Gestational age and lactation stage are important factors that affect DHM composition [3], impacting nutritional value, the concentration of biologically active components, such as hormones, lactoferrin [37], HM oligosaccharides [38], and the bacterial load [39]. This may imply that the infants’ outcomes could differ depending on whether the infants received milk from a mother who gave birth prematurely or at term.

Whereas our search retrieved no clinical data about the influence of the lactation stage of the donor, we were able to identify data about the influence of preterm compared with term DHM, with conflicting results.

However, in the study by Gross [22], milk from preterm donors was collected immediately postpartum, whereas milk from term donors was obtained several months into lactation. This design introduces 2 concurrent variables (gestational age and duration of lactation) that cannot be independently evaluated. Given the well-established changes in breast milk composition throughout lactation, these temporal differences in milk collection may represent a significant contributing factor to the reported growth outcomes [22].

In the 2 other studies, MOM was supplemented with preterm or term DHM, but data about the actual gestational age of the donor’s delivery were not given. Gialeli et al. [21] did not detail the absolute amount of DHM to MOM in the study, but the ratio was similar between groups. Protein intake was consistently higher in the preterm DHM group, which may be a chance finding since protein content of MOM was not a stratification criterion in this study.

In conclusion, the results of the available studies do not allow us to conclude conclusively whether the timing of donors’ delivery affects the outcomes of infants receiving DHM.

### Intervention 5 (donors characterized by different health status)

We did not identify any completed studies that evaluated the clinical impact of DHM-based on maternal health status. An ongoing study aims to evaluate the fecal microbiome composition of preterm infants fed HMOs secretor status-matched DHM (nonsecretors compared with secretors) [32]. HMOs are complex carbohydrates found uniquely in HM. They benefit preterm infants by promoting healthy gut bacteria, boosting immune function, and protecting against infections. The mother’s genetically determined secretor status is the main factor influencing HMO composition [40]. Because no data are currently available on the clinical impact of secretor status-matched DHM in preterm infants, this study could contribute new hypothesis-generating insights into how maternal genetic factors affect donor milk effectiveness.

### Intervention 8

#### Higher energy density DHM

Increasing the energy content of HM is recommended for avoiding nutritional deficiencies for the fast-growing premature infant. In the 3 included studies [25,27,30] increase in energy density was achieved either through HM evaporation (condensation) or by adding HM lyophilizate to the base HM. In our review, we excluded all studies using commercially available HM fortifiers due to the proprietary aspect of their preparation, which impedes comparisons [41,42]. Nars [27] developed a rationale to enrich DHM with DHM lyophilizate to reduce parenteral nutrition requirements, driven by the need to minimize fluid exposure and nutritional deficiencies in the absence of then available bovine milk-based multicomponent fortifiers. The 2 others eligible studies [25,30] aimed at avoiding bovine milk-based fortifiers. The contradictory data regarding weight gain between the studies may be attributed to changes in clinical practice over the extended time period during which these studies were conducted [25,27]. Safety data in those studies were very limited, and only 52 neonates were included in these comparisons.

#### Heat treatment studies

Heat treatment of HM for premature infants is often used to control microbial and viral colonization. However, the heat-dependent adverse effects of HoP on HM’s bioactive components are well documented, diminishing its beneficial properties and potentially impacting clinical outcomes. HoP at 62.5°C ± 0.5°C for 30 min represents the best available compromise. Meanwhile, alternative methods are being explored to reduce heat exposure to bioactive components while still ensuring microbial safety.

There is substantial preclinical data regarding alternatives to HoP and their respective impacts on HM [10]. In contrast, as we could show with our review, clinical evidence supporting the need for alternatives to HoP is very limited. Two historical observational studies found no difference in fat absorption rate and weight gain when DHM was boiled or sterilized [29,31]. Only 1 high-quality randomized clinical trial compares the clinical outcomes of premature infants whose MOM was supplemented with DHM subjected to different heat treatments [26].

#### Homogenization studies

Homogenization is a physical process, common in the dairy industry and for parenteral nutrition emulsions, aimed at dispersing mutually nonsoluble liquids to create a single uniform mixture [43]. Several techniques (e.g., sonication, blending, and high pressure) might be used, all aimed at disrupting the membrane surrounding milk fat globules to reduce and to uniform their size, increasing the surface available for digestive enzymes’ adsorption [24]. Interest in homogenization of DHM has recently emerged, in particular as a consequence of newly available DHM-derived products, including homogenized DHM-based fortifier [43]. According to these authors, although some compositional data suggest potential advantages of this processing technique, the literature assessing the benefits of an exclusive HM diet is limited to nonhomogenized HM-based products. In preclinical studies on bovine milk-derived products, homogenization-induced changes to the physical and chemical properties of the milk fat globules alter their interactions with digestive enzymes, including gastric and pancreatic lipases, and render them more susceptible to digestion in the stomach [44,45]. However, purported clinical benefits, such as increased fat absorption and subsequent weight gain improvements, or reduced fat loss during tube feeding, have not been demonstrated to date.

In our review, we identified 2 clinical trials that compared clinical outcomes of premature infants fed ultrasonic homogenized compared with nonhomogenized DHM. The authors observed an increased net weight gain and an increase in gastric lipolysis, an increase in gastric volume, and a slower gastric emptying rate when feeding homogenized DHM compared with a control group. However, interpreting these data remains challenging [9]. It is unclear whether higher gastric lipolysis can enhance lipolysis and absorption in the intestinal phase. Although the reduced gastric emptying rate of homogenized DHM could potentially increase the risk of feeding intolerance and gastrointestinal infections, this must be considered in the context of the much longer gastric half-emptying times observed in infants fed BMS [44]. Only 8 infants were analyzed in this study, and available clinical evidence on the benefits of homogenized DHM is based on 38 infants.

### Limitations and strengths

We included manuscripts that had at least an English abstract. During the search process, we recognized that this criterion might have excluded historical research, particularly from countries with an established history in HM banking, such as Germany, Austria, France, and Brazil who did not include an English abstract but published only in their respective native language. Even though this historical research may not have met the current quality standards required to draw reliable conclusions and inform today’s clinical practice, this aspect remains to be considered. Another limitation is the overall low number of available studies, with several relying on very small samples and some being older, resulting in lower quality assessment scores. The strength of this systematic review includes an extensive literature search, rigorous inclusion criteria, and robust data synthesis methods performed by a multidisciplinary team of healthcare professionals, researchers, social scientists, and health information professionals with comprehensive expertise in all aspects of HM banking. Nonlimiting to publication in English, manually searching the reference lists, and including ongoing studies, should reduce publication bias and publication lag. However, studies without English abstracts may be missing from this review.

In conclusion, we systematically reviewed and mapped the available evidence for clinical health and growth outcomes in premature infants when different interventions for DHM were applied. Our analysis revealed a significant paucity of clinical data for several interventions and a complete lack of evidence for most of our research questions, indicating insufficient evidence to support the clinical efficacy of these interventions. It is crucial to address these evidence gaps, as the interventions we analyzed are currently being integrated into clinical practice without sufficient knowledge regarding their potential benefits or harm for preterm infants. Conversely, preterm infants risk presently receiving suboptimal treatment if these interventions are proven to be effective in clinical trials. A cautious, incremental approach may be warranted when testing interventions in a vulnerable population such as premature infants. However, those preclinical or piloting clinical trials should aim to establish a viable pathway for future clinical DHM trials. These adequately powered clinical trials should then focus on easily reproducible and comparable outcomes based on sound physiological principles with clinical outcomes that are meaningful to healthcare professionals, parents, and foremost the infants themselves [46].

## Author contributions

The authors’ responsibilities were as follows – AW, SG, DK: conceptualized the manuscript; AW, MB-A: supervised and administered the project; AW, SG, AB-J, DK: acquired funding; MG, CS, TC: curated the data; TC, BW: performed the formal analysis; AB-J, DK, MG: prepared the data visualization; and all authors: designed the review methodology, conducted the investigation, critically reviewed and commented on the manuscript, agreed to be accountable for the final manuscript, and read and approved the final manuscript.

## Funding

The research questions involved in this systematic review were provided by the WHO guidelines group, and this study was performed with the guidance of the WHO Department of Nutrition and Food Safety, following their “Call for authors—Systematic reviews on donor human milk banking processes.”

## Conflict of interest

LC and MG are coinventors of an EU patent (EP 15176792.8-1358—continuous flow pasteurizer for small amounts of liquid foods) concerning an high-temperature short-time pasteurizer for small volumes of liquid foods. A prototype has been developed and validated for treating human milk. The patent has been licensed to Labor Baby S.r.l, an Italian company that has recently produced a definitive device. The patent is owned by the Italian National Research Council, the University of Turin, and Giada s.a.s. AW is a coinvestigator of the Polish patent number Pat.238537, submission number P.429126 concerning the optimization of high-pressure preservation of human milk, which was created within the framework of the uncommercial research carried out based on the project financed by the National Centre for Research and Development for nongovernmental organizations. The leader of the project “Lactotechnology as a response for vulnerable babies” was the Human Milk Bank Foundation, and a member of the consortium was Warsaw Medical University and 4 other entities. The present conflicts of interest have been previously disclosed to the other members of the expert group, and a consensus on its mitigation strategy has been reached. As participants to the review team, LC, MG, and AW are engaged in abstaining from participation in discussion, drafting, and reporting of the evidence that will be found pertaining to the use of different pasteurization methods on human milk. All other authors report no conflicts of interest.
