# Supplementary material for: A Systematic Review on the Impact of Donor Characteristics and Donor Milk Handling on Infant Health and Growth
Source: Adv Nutr. 2026 Feb 19;17(4):100609. doi: 10.1016/j.advnut.2026.100609 (PMC13068549; doi:10.1016/j.advnut.2026.100609)
Supplement: Multimedia component 1 [file mmc1.pdf]

Impact of Donor Characteristics and Donor Milk Handling on Infant Health and Growth Outcomes – A Systematic Review - Supplementary Material

Daniel Klotz, Agnieszka Bzikowska-Jura

Supplementary table 1: Prisma checklist

PRISMA-P (Preferred Reporting Items for Systematic review and Meta-Analysis Protocols) 2015 checklist: recommended items to address in a systematic review protocol*

Section and topic	Item No	Checklist item	Page Number
**ADMINISTRATIVE INFORMATION**			
Title:			
Identification	1a	Identify the report as a protocol of a systematic review	1
Update	1b	If the protocol is for an update of a previous systematic review, identify as such	n.a.
Registration	2	If registered, provide the name of the registry (such as PROSPERO) and registration number	5
Authors:			
Contact	3a	Provide name, institutional affiliation, e-mail address of all protocol authors; provide physical mailing address of corresponding author	1
Contributions	3b	Describe contributions of protocol authors and identify the guarantor of the review	17-18
Amendments	4	If the protocol represents an amendment of a previously completed or published protocol, identify as such and list changes; otherwise, state plan for documenting important protocol amendments	n.a.
Support:			
Sources	5a	Indicate sources of financial or other support for the review	18
Sponsor	5b	Provide name for the review funder and/or sponsor	18
Role of sponsor or funder	5c	Describe roles of funder(s), sponsor(s), and/or institution(s), if any, in developing the protocol	18
**INTRODUCTION**			
Rationale	6	Describe the rationale for the review in the context of what is already known	3
Objectives	7	Provide an explicit statement of the question(s) the review will address with reference to participants, interventions, comparators, and outcomes (PICO)	4/table 1
**METHODS**			
Eligibility criteria	8	Specify the study characteristics (such as PICO, study design, setting, time frame) and report characteristics (such as years considered, language, publication status) to be used as criteria for eligibility for the review	4-6
Information sources	9	Describe all intended information sources (such as electronic databases, contact with study authors, trial registers or other grey literature sources) with planned dates of coverage	5
Search strategy	10	Present draft of search strategy to be used for at least one electronic database, including planned limits, such that it could be repeated	Online Supplement/4
Study records:			
Data management	11a	Describe the mechanism(s) that will be used to manage records and data throughout the review	5
Selection process	11b	State the process that will be used for selecting studies (such as two independent reviewers) through each phase of the review (that is, screening, eligibility and inclusion in meta-analysis)	5
Data collection process	11c	Describe planned method of extracting data from reports (such as piloting forms, done independently, in duplicate), any processes for obtaining and confirming data from investigators	5

Data items	12	List and define all variables for which data will be sought (such as PICO items, funding sources), any pre-planned data assumptions and simplifications	4/Table1
Outcomes and prioritization	13	List and define all outcomes for which data will be sought, including prioritization of main and additional outcomes, with rationale	4
Risk of bias in individual studies	14	Describe anticipated methods for assessing risk of bias of individual studies, including whether this will be done at the outcome or study level, or both; state how this information will be used in data synthesis	6-7
Data synthesis	15a	Describe criteria under which study data will be quantitatively synthesised	
	15b	If data are appropriate for quantitative synthesis, describe planned summary measures, methods of handling data and methods of combining data from studies, including any planned exploration of consistency (such as I^2, Kendall's τ)	n.a.
	15c	Describe any proposed additional analyses (such as sensitivity or subgroup analyses, meta-regression)	n.a.
	15d	If quantitative synthesis is not appropriate, describe the type of summary planned	7
Meta-bias(es)	16	Specify any planned assessment of meta-bias(es) (such as publication bias across studies, selective reporting within studies)	6-7
Confidence in cumulative evidence	17	Describe how the strength of the body of evidence will be assessed (such as GRADE)	6-7

* It is strongly recommended that this checklist be read in conjunction with the PRISMA-P Explanation and Elaboration (cite when available) for important clarification on the items. Amendments to a review protocol should be tracked and dated. The copyright for PRISMA-P (including checklist) is held by the PRISMA-P Group and is distributed under a Creative Commons Attribution Licence 4.0.

Impact of Donor Characteristics and Donor Milk Handling on Infant Health and Growth Outcomes – A Systematic Review - Supplementary Material

Daniel Klotz, Agnieszka Bzikowska-Jura

Supplementary table 2: Search strategy (example for Ovid MEDLINE)

	**Searched terms**	**Results**
1	((milk or breastmilk or breastfe* or lactat*) adj3 (donat* or donor* or bank* or center* or centre* or share* or sharing)).ti;ab;kf.	2663
2	Milk; Human/ or Breast Feeding/ or exp Lactation/	102269
3	(donat* or donor* or bank* or center* or centre* or share* or sharing).ti;ab;kf.	2036665
4	2 and 3	5690
5	Milk Banks/	723
6	1 or 4 or 5	6601
7	exp Infant/	1268879
9	(Outcome* or impact* or affect* or influenc* or result* or consequenc* or caus* or issue* or threat* or effect* or benefit* or harm* or risk factor* or tolera* or growth* or development* or neurodevelop* or behavio* or weight or digest* or diet or nutrition or malnutrition or deficien* or illness* or death* or morbidity or mortality or infection* or transmi* or sepsis* or enterocolitis* or cytomegalovirus* or HIV or immunologic or diarrh* or meningitis or urinary tract infection* or UTI or Expos* or Quality or Complication*).ti;ab;kf.	23603878
10	7 and 8	854937
11	((infant* or baby or babies or neonate* or new born* or newborn* or preterm or pre-term or child*) adj4 (Outcome* or impact* or affect* or influenc* or result* or consequenc* or caus* or issue* or threat* or effect* or benefit* or harm* or risk factor* or tolera* or growth* or development* or neurodevelop* or behavio* or weight or digest* or diet or nutrition or malnutrition or deficien* or illness* or death* or morbidity or mortality or infection* or transmi* or sepsis* or enterocolitis* or cytomegalovirus* or HIV or immunologic or diarrh* or meningitis or urinary tract infection* or UTI or Expos* or Quality or Complication*)).ti;ab;kf.	706282
12	((infant* or baby or babies or neonate* or new born* or newborn* or preterm or pre-term or child*).ti;ab;kf. or exp *Infant/) and exp *"Growth and Development"/	79301
13	9 or 10 or 11	1296223
14	6 and 12	3752
15	13 not (Animals/ not (Animals/ and Humans/))	3737
	**Total Records downloaded**	**3737**

Impact of Donor Characteristics and Donor Milk Handling on Infant Health and Growth Outcomes – A Systematic Review - Supplementary Material

Daniel Klotz, Agnieszka Bzikowska-Jura

Supplementary table 3: Quality Assessment of randomised controlled trials

Study	Methodology												
	Did the study address a clearly focused research question?	Are the inclusion criteria clearly described?	Was the method of randomization appropriate?	Was randomization sufficient to eliminate systematic bias?	Was the allocation sequence concealed from investigators and participants?	Were losses to follow-up and exclusions after randomization accounted for?	Were participants analyzed in the study groups to which they were randomized (intention-to-treat analysis)?	Was the study stopped early? If so, what was the reason?*	Were the participants (mothers, caregivers) 'blind' to the intervention they were given?	Were the investigators 'blind' to the intervention they were giving to participants?	Were the people assessing/analyzing outcome/s 'blinded'?	Were the study groups similar at the start of the randomized controlled trial?	Apart from the experimental intervention, did each study group receive the same level of care (that is, were they treated equally)?	Overall score
Azad 2019[32]	2	2	2	2	2				0	0	2			12/16
de Oliveira 2017[24]	2	2	2	2	2	2	2	2	2	2	2	2	2	26/26
dos Santos 2007[25]	2	2	2	2	0	0	2	2	0	0	0	2	2	16/26
Garcia-Lara 2024[26]	2	2	2	2	2	2	2	2	2	2	2	2	2	26/26
Gialeli 2023[21]	2	2	2	2	2		2	2	2	2	0	2	2	22/24
Gross 1983[22]	2	2	2	2	2	0	0	2	0	0	0	0	2	14/26
Hemati 2024[33]	2	2	2	2	2	1	1		1	1	1	1		16/22
Rayol 1993[28]	2	2	2	2	2	1	2	2	0	0	0	2	2	19/26
Soni 2022[23]	2	2	2	2	0	0	2	2	2	0	0	2	2	18/26
Thomaz 2014[30]	2	2	2	0	0	1	0	2	0	0	0	2	2	13/26
Williamson 1978[31]	2	0			1			2	1	0	1	2	1	10/18
Soderhjelm 1952[29]	2	2						2	1	1	1	1	2	12/16

2 stands for 'Yes', 1 for 'I can't tell' 0 for 'No'. Empty cells stand for not applicable

* 2 stands for 'No', 1- 'I can't tell', 0 for 'Yes'

Study	Results										
	Was a power calculation undertaken?	Were the outcomes clearly specified?	Were the effects reported?	Were the results reported for each outcome in each study group at each follow-up interval?	Was there any missing or incomplete data? *	Was there a differential drop-out between the study groups that could affect the results? *	Were potential sources of bias identified?	Were appropriate statistical tests used?	Were p values reported?	Was the precision of the estimate of the intervention or treatment effect reported? Were confidence intervals (CIs) reported?	Overall score
Azad 2019[32]											0/0
de Oliveira 2017[24]	2	2	0	2	0	0	0	2	2	0	10/20
dos Santos 2007[25]	0	2	0	0	2	2	0	2	2	2	12/20
Garcia-Lara 2024[26]	2	2	2	2	2	2	0	2	2	0	16/20
Gialeli 2023[21]	0	2	0	2	2	2	0	2	2	0	12/20
Gross 1983[22]	0	2	0	2	2	2	0	2	2	0	12/20
Hemati 2024[33]											0/0
Rayol 1993[28]	0	2	0	0	2	2	0	2	2	0	10/20
Soni 2022[23]	0	2	0	2	2	2	0	2	2	2	14/20
Thomaz 2014[30]	0	2	0	0	2	2	0	2	2	0	10/20
Williamson 1978[31]	0	1	0				0	2	2	0	5/14
Soderhjelm 1952[29]	0	2	0	2	2	1	0	0	0	0	7/20

2 stands for ‘Yes’, 1 for ‘I can't tell’ 0 for ‘No’. Empty cells stand for not applicable
* 2 stands for ‘No’, 1- ‘I can't tell’, 0 for ‘Yes’

Impact of Donor Characteristics and Donor Milk Handling on Infant Health and Growth Outcomes – A Systematic Review - Supplementary Material

Daniel Klotz, Agnieszka Bzikowska-Jura

Supplementary table 4: Quality Assessment Observational Studies

Study	Methodology									Overall score
	Did the study address a clearly focused research question?	Are the inclusion criteria clearly described?	Are the sources and methods of case ascertainment and control selection clearly described?	Are methods of follow up clearly described?	Are matching criteria clearly described?	Are variables identified and clearly described?	Is the sample relevant to the research goals?	Sample recruitment: random	Are the measurement methods relevant to the the research goals?	
Nars 1984[27]	2	0	1	2	0	2	2	0	2	11/20
Perry 2024[34]	2	2		1	1	1	1	0	1	9/18
Soderhjelm 1952[29]	2	0		1	0	2	2	0	2	11/18
Williamson 1978[31]	2	0	1		0	0	0	0	2	11/20

2 stands for 'Yes', 1 for 'I can't tell' 0 for 'No'. Empty cells stand for not applicable
* 2 stands for 'No', 1- 'I can't tell', 0 for 'Yes'

Study	Results													
	Was a sample power calculation undertaken?	Were the outcomes clearly specified?	Were the effects reported ?	Were the results reported for each outcome in each study group at each stage (if applicable)?	Was there any missing or incomplete data?*	Were the missing data addressed (explained , estimated) ?	Was there a differential drop-out between the measurem ents (if applicable) ?	Were potential sources of bias identified?	Were appropriat e statistical tests used?	Were p values reported?	Was the precision of the estimate of the intervention or treatment effect reported? Were confidence intervals (CIs) reported?	Is the participants characteristics provided?	Was the follow-up time summarized ?	Overall score
Nars 1984[27]	0	2	0	0	1			0	0	0	0	0	0	3/22
Perry 2024[34]	0													0/2
Soderhjelm 1952[29]	0	2	0	0	1			0	0	0	0	0	0	3/22
Williamson 1978[31]	0	2	0	0	1			0	2	2	0	2	0	9/22

2 stands for 'Yes', 1 for 'I can't tell' 0 for 'No'. Empty cells stand for not applicable
* 2 stands for 'No', 1- 'I can't tell', 0 for 'Yes'

Impact of Donor Characteristics and Donor Milk Handling on Infant Health and Growth Outcomes – A Systematic Review - Supplementary Material
Daniel Klotz, Agnieszka Bzikowska-Jura

Supplementary figure 1: Quality Assessment Workflow

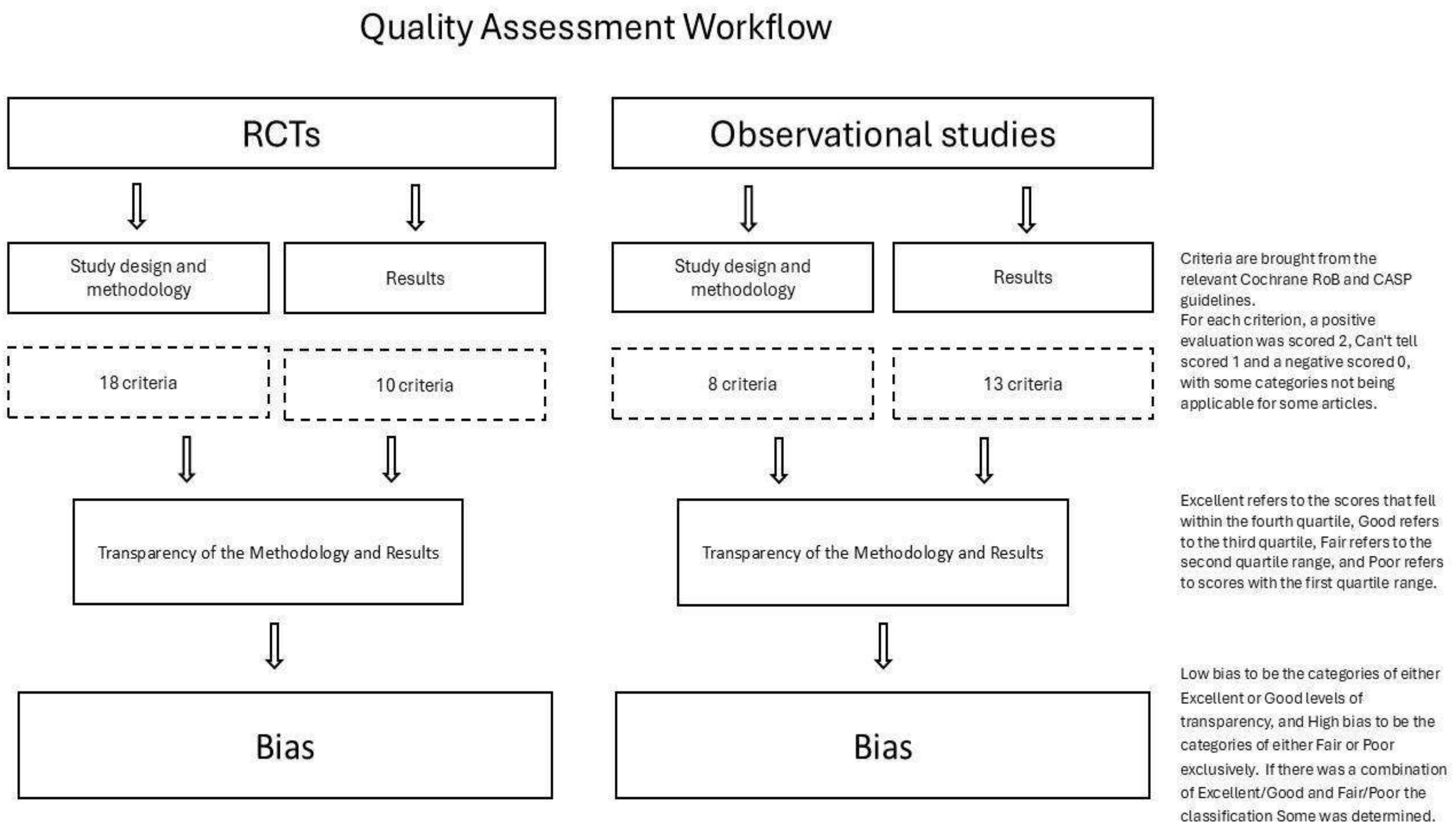

Supplementary Figure 2: Overall Table of Quality Assessment results for included studies.

	Transparency of the methodology	Transparency of the results	Bias	
Azad et al., 2019[32]	Good	NA	Low	▲
deOliveira 2017[24]	Excellent	Fair	Low	■
dosSantos 2007[25]	Good	Good	Low	■
Garica-Lara 2024[26]	Excellent	Excellent	Low	■
Gialeli 2023[21]	Excellent	Good	Low	■
Gross 1983[22]	Good	Good	Low	■
Hemati et al., 2024[33]	Good	NA	Low	▲
Rayol 1993[28]	Good	Fair	Some	■
Soni 2022[23]	Good	Good	Low	■
Thomaz 2014[30]	Fair	Fair	High	■
Nars 1984[27]	Good	Poor	Some	●
Perry et al., 2024[34]	Good	NA	Low	▲
Soderhjelm, 1952[29]	Good	Poor	Some	●
Williamson et al., 1978[31]	Good	Fair	Some	●

□ RCT ○ Observational study Δ On-going study. The Transparency of the Methodology and Results is grouped around the categorical classifications of Excellent refers to the scores that fell within the fourth quartile, Good refers to the third quartile, Fair refers to the second quartile range, and Poor refers to scores within the first quartile range regarding transparency. Regarding the Bias classification, we determinized Low (green) bias to be the category of either Excellent or Good levels of transparency, and High (red -) bias to be the category of either Fair or Poor exclusively. If there was a combination of Excellent/Good and Fair/Poor the classification Some (yellow) was determined. The complete scores can be viewed in Appendix 1,2,3,4 where 'Yes' was scored 2, 'I can't tell' scored 1 and 'No' scored 0, with some categories not being applicable for some articles. Quartiles were determined from each of these articles using these scores. *Study is listed as completed in a trial registry but results are not published.

Impact of Donor Characteristics and Donor Milk Handling on Infant Health and Growth Outcomes – A Systematic Review - Supplementary Material

Daniel Klotz, Agnieszka Bzikowska-Jura

Supplementary table 5: Overall GRADE

Intervention group	Outcome under consideration	Relative importance of the outcome (1-9)	Studies under consideration	Design	Risk of Bias	Inconsistency	Indirectness	Imprecision	Other (Publication Bias)	Certainty (overall score)
Preterm vs term	Growth	9	21-23	3 RCT	Not at all serious RoB	Not really serious inconsistency	Not at all serious indirectness	Serious imprecision	Not at all serious	High Certainty
Preterm vs term	Mortality	9	21,23	2 RCT	Not at all serious RoB	Not really serious inconsistency	Not at all serious indirectness	Serious imprecision	Not at all serious	High Certainty
Preterm vs term	Morbidity	9	21,23	2 RCT	Not at all serious RoB	Not really serious inconsistency	Not at all serious indirectness	Serious imprecision	Not at all serious	High Certainty
Preterm vs term	Feeding tolerance	8	23	1 RCT	Not at all serious RoB	Not applicable (1 study only)	Not at all serious indirectness	Serious imprecision	Not at all serious	Moderate Certainty
Preterm vs term	Other infections	9	21,23	2 RCT	Not at all serious RoB	Not really serious inconsistency	Not serious indirectness	Serious imprecision	Not at all serious	High Certainty
Preterm vs term	Nutrient deficiencies	6	22,23	2 RCT	Not at all serious RoB	Serious inconsistency	Serious indirectness	Serious imprecision	Not at all serious	Moderate Certainty
Homogenization	Growth	9	28	1 RCT	Serious RoB	Not applicable (1 study only)	Not at all serious Indirectness	Serious imprecision	Not at all serious	Low Certainty
Concentration/fort ification	Growth	9	24,26	1 RCT, 1 Obs	Not really serious RoB	Not really serious inconsistency	Not serious indirectness	Not Serious imprecision	Not at all serious	Moderate Certainty
Concentration/fort ification	Morbidity	9	26	1 Obs	Serious RoB	Not applicable (1 study only)	Not at all serious indirectness	Serious imprecision	Not at all serious	Very Low Certainty
Concentration/fort ification	Mortality	9	26	1 Obs	Serious RoB	Not applicable (1 study only)	Not serious Indirectness	Serious imprecision	Not at all serious	Very Low Certainty
Concentration/fort ification	Feeding tolerance	8	26	1 Obs	Serious RoB	Not applicable (1 study only)	Very serious indirectness	Serious imprecision	Not at all serious	Very Low Certainty
Concentration/fort ification	Nutrient deficiencies	6	24,26,30	2 RCT, 1 Obs	Serious RoB	Not really serious inconsistency	Not at all serious indirectness	Not Serious imprecision	Not at all serious	Moderate Certainty
Heat treatment	Growth	9	25,29,31	1 RCT, 2 Obs	Serious RoB	Serious inconsistency	Not serious indirectness	Serious imprecision	Not at all serious	Low Certainty
Heat treatment	Mortality	9	25	1 RCT	Not at all serious RoB	Not applicable (1 study only)	Not at all serious Indirectness	Serious imprecision	Not at all serious	Moderate Certainty
Heat treatment	Morbidity	9	25	1 RCT	Not at all serious RoB	Not applicable (1 study only)	Not at all serious indirectness	Serious imprecision	Not at all serious	Moderate Certainty
Heat treatment	Feeding tolerance	8	25	1 RCT	Not at all serious RoB	Not applicable (1 study only)	Serious indirectness	Serious imprecision	Not at all serious	Low Certainty
Heat treatment	Other infections	9	25	1 RCT	Not at all serious RoB	Not applicable (1 study only)	Not at all serious Indirectness	Serious imprecision	Not at all serious	Moderate Certainty
Heat treatment	Nutrient deficiencies	6	31	1 Obs	Serious RoB	Not applicable (1 study only)	Not serious indirectness	Serious imprecision	Not at all serious	Very Low Certainty
Heat treatment	Breastfeeding	9	25	1 RCT	Not at all serious RoB	Not applicable (1 study only)	Not serious Indirectness	Serious imprecision	Not at all serious	Moderate Certainty

Risk of Bias was determined by the Quality Assessment details for each study involved. Inconsistency and Indirectness were determined by two clinical team members and the other categories involved a concensus between two methodologists in the team. Following EPOC, RCTs began with a score of 4 and observational studies began with a score of 2. If a dimension was listed at either 'not applicable' or 'not at all serious' no change occurred. If it was determined to be 'not really serious' one point was removed, and if determined to be 'serious' two points were removed. The overall final 'Certainty' labels in the final column are based on these scores and are 'high certainty' (meaning a grading of 'not very serious' across all dimensions, resulting in reporting very confident that the true effect is close to the estimate), 'moderate certainty' (meaning a grading of 'not very' or 'serious' on one dimension, resulting in moderate confidence), 'low certainty' (meaning a grading of 'not very serious' or serious' on two dimensions resulting limited confidence), and 'very low certainty' meaning a grading of 'not very serious' or 'serious' on three or more dimensions, resulting in very little confidence in the effect estimate the true effect).

Impact of Donor Characteristics and Donor Milk Handling on Infant Health and Growth Outcomes – A Systematic Review - Supplementary Material

Daniel Klotz, Agnieszka Bzikowska-Jura

Supplementary table 6: Excluded studies with reason for exclusion

**Authors**	**Year**	**Title**	**DOI**	**Reason for exclusion**
Adhisivam; B; Kohat; D; Tanigasalam; V; Bhat; V; Plakkal; N; Palanivel; C	2019	Does fortification of pasteurized donor human milk increase the incidence of necrotizing enterocolitis among preterm neonates? A randomized controlled trial	10.1080/14767058.2018.1461828	Wrong intervention – effect of fortification with commercial product
Aprile; MM; Feferbaum; R; Andreassa; N; Leone; C	2010	Growth of very low birth weight infants fed with milk from a human milk bank selected according to the caloric and protein value	10.1590/s1807-59322010000800002	Wrong intervention – own mother's milk vs DHM
Bedwell; SM; Buster; B; Sekar; K	2021	The effect of a continuous milk warming system on weight gain in very low birth-weight infants: a randomized controlled trial	10.1097/ANC.0000000000000818	Wrong intervention – own mother's milk and DHM are used; and no separate analysis for each is provided
Boehm; G; Müller; DM; Senger; H; Borte; M; Moro; G	1993	Nitrogen and fat balances in very low birth weight infants fed human milk fortified with human milk or bovine milk protein	10.1007/BF01956152	Wrong intervention – effect of fortification with commercial products
Clifford; V; Klein; LD; Brown; R; Sulfaro; C; Hoad; V; Gosbell; IB; Pink; J	2022	Donor and recipient safety in human milk banking	10.1111/jpc.16066	Wrong intervention - adverse events connected with milk donation
Dougherty; D	2011	There is an absence of randomised trials investigating the use of preterm banked milk compared to term banked milk for very low birthweight infants.	10.1136/ebn1109	Wrong publication type - commentary
Fu; TT; Schroder; PE.; Poindexter; BB	2019	Macronutrient analysis of target-pooled donor breast milk and corresponding growth in very low birth weight infants	10.3390/nu11081884	Wrong intervention – commercially purchased DHM labelled for calorific value
Hair AB; Blanco CL; Hawthorne KM; Moreira A.; Lee ML; Rechtman DJ; Abrams SA	2014	Human milk cream enhances growth when supplementing standard fortification of an exclusive human milk-based diet in VLBW infants	10.1136/archdischild-2014-307384.191	Wrong publication type - abstract
Hung; HY; Hsu; YY; Chang; YJ	2013	Comparison of physiological and behavioral responses to fresh and thawed breastmilk in premature infants--a preliminary study	10.1089/bfm.2012.0026	Wrong intervention – own mother's milk is used
Jordan-Crowe S; Dorcin M; Thompson C; Wilson-Costello D; Madden J	2020	Comparison of growth and morbidities in extremely low gestational age infants fed sterilized shelf-stable versus holder pasteurized donor human milk	10.1089/bfm.2020.29162.abstracts	Wrong publication type - abstract
Kokinopoulos; D; Photopoulos; S; Varvarigou; N; Kafegidakis; L; Xanthou; M	1991	The effect of human milk; protein-fortified human milk and formula on immunologic factors of newborn infants		Wrong publication type - book chapter
Mali; V; Patki; S; Patil; U	2019	Human milk component administration in preterm neonates: a randomised controlled trial	10.1097/MPG.0000000000002403	Wrong publication type - abstract

Authors	Year	Title	DOI	Reason for exclusion
Mangalapally N; Patel S; Vlk AM; Capriolo C; Schofield EE; Davis NL	2022	Outcomes of donor breast milk and Prolacta use in an urban level IV neonatal intensive care unit		Wrong publication type - abstract
Mills; L; Chappell; KE; Emsley; R; Alavi; A; Andrzejewska; I; Santhakumaran; S; Nicholl; R; Chang; J; Uthaya; S; Modi; N	2023	Preterm formula; fortified or unfortified human milk for very preterm infants; the PREMFOOD study: a parallel randomised feasibility trial	10.1159/000535498	Wrong intervention – effect of fortification with commercial product
Patra; K; Greene; MM; Tobin; G; Casini; G; Esquerra-Zwiers; AL; Meier; PP; Patel; AL	2022	Neurodevelopmental outcome in very low birth weight infants exposed to donor milk.	10.1055/s-0040-1722597	Wrong intervention - no different DHM
Perrella; SL; Hepworth; AR; Gridneva; Z; Simmer; KN; Hartmann; PE; Geddes; DT	2015	Gastric emptying and curding of pasteurized donor human milk and mother's own milk in preterm infants	10.1097/MPG.0000000000000776	Wrong intervention – effect of fortification with commercial product
Polberger; SK; Axelsson; IE; Räihä; NC	1990	Amino acid concentrations in plasma and urine in very low birth weight infants fed protein-unenriched or human milk protein-enriched human milk		Wrong intervention – own mother's milk and DHM are used; and no separate analysis for each is provided
Polberger; S; Räihä; NC; Juvonen; P; Moro; GE; Minoli; I; Warm; A	1999	Individualized protein fortification of human milk for preterm infants: comparison of ultrafiltrated human milk protein and a bovine whey fortifier	10.1097/00005176-199909000-00017	Wrong intervention – own mother's milk and DHM are used; and no separate analysis for each is provided
Quan; M; Wang; D; Gou; L; Sun; Z; Ma; J; Zhang; L; Wang; C; Schibler; K; Li; Z	2020	Individualized Human Milk Fortification to Improve the Growth of Hospitalized Preterm Infants	10.1002/ncp.10366	Wrong intervention – effect of fortification with commercial product; only own mother's milk
Raban; S; Santhakumaran; S; Keraan; Q; Joolay; Y; Uthaya; S; Horn; A; Modi; N; Harrison; M	2016	A randomised controlled trial of high vs low volume initiation and rapid vs slow advancement of milk feeds in infants with birthweights ≤ 1000 g in a resource-limited setting	10.1179/2046905515Y.0000000056	Wrong intervention – own mother's milk and DHM are used; and no separate analysis for each is provided
Ramos-Garcia V; Ten-Domenech I; Moreno-Gimenez A; Campos-Berga L; Parra-Llorca A; Ramon-Beltran A; Vaya MJ; Mohareb F; Molitor C; Refinetti P; Silva A; Rodrigues LA; Rezzi S; Hodgson A C C; Canarelli S; Bathrellou E; Mamalaki E; Karipidou M; Poulimeneas D; Yannakoulia M; Akhgar C K; Schwaighofer A; Lendl B; Karrer J; Migliorelli D; Generelli S; Gormaz M; Vasileiadis M; Kuligowski J; Vento M	2023	Fact-based nutrition for infants and lactating mothers - The NUTRISHIELD study	10.3389/fped.2023.1130179	Wrong intervention - no different DHM
Rodriguero; CB; Ichisato; SMT.; De Oliveira Trombelli; FS; MacEdo; V; De Oliveira; MLF.; Rossetto; EG	2019	Premature infants fed with human milk versus human milk enhanced with FM85®	10.1590/1982-0194201900075	Wrong intervention – effect of fortification with commercial product
Tabasso; C; Piemontese; P; Pesenti; N; Perrone; M; Menis; C; Liotto; N; Amato; O; Orsi; A; Mallardi; D; Mosca; F; Roggero; P	2023	Pooling strategies to modify macronutrient content of pasteurized donor human milk.	10.1089/bfm.2023.0043	Wrong outcomes - no infant outcome; effect on milk composition
Tanaka; A; Rugolo; LMSS; Miranda; AFM; Trindade; Cleide EP	2006	Fractional sodium excretion; urinary osmolality and specific gravity in preterm infants fed with fortified donor human milk	10.2223/JPED.1504	Wrong intervention – effect of fortification with commercial product
Valentine; CJ; Morrow; G; Pennell; M; Morrow; AL; Hodge; A; Haban-Bartz; A; Collins; K; Rogers; LK	2013	Randomized controlled trial of docosahexaenoic acid supplementation in midwestern U.S. human milk donors	10.1089/bfm.2011.0126	Wrong outcomes - no infant outcome; results are estimation of infants' intake

Authors	Year	Title	DOI	Reason for exclusion
NCT04640805	2020	Targeted fortification of pasteurized donor human milk		Wrong intervention – effect of fortification with commercial products
CTRI/2020/02/023569	2020	Comparison of supplementation of preterm versus term donor pooled human milk on growth of very low birth weight neonates		Wrong publication type - protocol of one study published and included
NCT01626508	2012	Prospective Evaluation in Older Premature, Untreated Breast Milk and Milk Processed by the Breast-milk Bank (ADiLL)		Wrong outcomes - no infant outcome; effect on milk composition
